# Universal health coverage in Cambodia: current status and future prospects

**DOI:** 10.7189/jogh.15.03016

**Published:** 2025-03-07

**Authors:** Virak Sorn

**Affiliations:** Faculty of Health Science and Biotechnology, University of Puthisastra, Phnom Penh, Cambodia

## Abstract

Despite improvements in health indices over the past decades, Cambodia faces significant challenges in achieving universal health coverage (UHC). With an effective UHC score of 58 in 2021 – lower than regional neighbours – the country struggles with disparities in health care access, urban-rural inequalities, and rising non-communicable diseases. The health care system continues to be heavily privatised, with out-of-pocket charges accounting for 55% of overall health expenditures in 2021. Although they have increased financial protection, government programs like the National Social Security Fund and Health Equity Funds are still insufficient. Strengthening public health care, decentralising governance, adopting digital health solutions, and establishing a Public Health Commission are crucial steps toward an equitable and effective UHC by 2035.

Universal health coverage (UHC) in Cambodia is still many years away, despite the country’s health indices improving over time. While Cambodia has made progress in the last thirty years, its effective UHC score of 58 in 2021 still places it lower than that of its neighbours, Vietnam (score 68), Thailand (score 82), and emerging economy peers, Singapore (score 89), Korea (score 89), Japan (score 83), and China (score 81), with East Asia and Pacific as a whole scoring 75 [1]. The overall fertility rate is 2.34 children per woman; the infant and maternal mortality rates are 20.17 for 1000 live births and 218 per 100 000 live births. The life expectancy is 70.74 years; however, there are significant variations according to caste, area, gender, class, and geography [2–4]. The health care system in Cambodia faces challenges from urbanisation, socioeconomic inequality, and limited access to high-quality health care [5]. The increased occurrence of non-communicable diseases in Cambodian society is a result of dietary trends that have shifted toward sugar, salt, fat, and alcohol [6,7]. These diseases can cause addiction and mental health issues [7]. The majority of health care workers are concentrated in cities, with two-fourths of doctors and three-quarters of specialists working in Phnom Penh, the country’s capital, even though nearly three quarters of the population lives in both rural and urban areas [5,8]. Given these issues, the government should prioritise two goals – raising the standard of health care delivered and expanding access to health care services for all Cambodians [9].

Recently, Cambodia’s health care system has comprised both the public and private sectors. There are three levels to the public health care system: the central, provincial, and operational district levels. Each level within the health system has a designated role and function [9]. The central level includes the various Ministry of Health departments, training institutions, national centres, and eight national hospitals. The provincial level is the interface between the central and operational district levels, with 25 provincial health departments. The operational district level is the most peripheral subunit within the health system and the one closest to the population, which is composed of 92 district referral hospitals, 1222 health centres, and 128 community health posts at the commune [10]. About 20% of Cambodia’s total health market is served by public hospitals and health centres that focus on the low-income group, while the private sector serves the remaining 80% of the health care market through private hospitals, clinics, and pharmacies. In practical terms, private clinics typically offer outpatient curative consultations, while the public sector provides most preventive services and inpatient care [9,10]. However, the private sector saw a significant increase this recent time, which accounts for up to three quarters of primary care consultations in urban and rural areas. The rise of private clinics means a rise in out-of-pocket costs as well; however, some private clinics are ineffective in treating diseases, which is somehow caused to serve conditions [11,12]. According to the issue raised above, the government will need to work really hard in order to address those two issues effectively. Based on expectations of sustained economic development, the government intends to raise spending, as outlined in the Health Strategic Plan 2016–20, focused on two main tasks – the Health Information System Master Plan, aiming to address weaknesses in health care data quality, and the Health Workforce Development Plan, aiming to fill an expected shortfall in health care workers [9].

Despite obstacles like high out-of-pocket costs and a loosely regulated private sector, the Cambodian government is dedicated to enhancing health care access and quality. The impoverished can protect their finances from risk through programs like Health Equity Funds (HEF), and voucher schemes provide financial risk protection for the poor [11–15]. However, there are still issues with health care service quality and access, which emphasises the need for ongoing initiatives to improve health care access. According to the report from Deutsche Gesellschaft für Internationale Zusammenarbeit GmbH Cambodia in 2021, out-of-pocket payments in Cambodia represented 63% in 2017 [9] and slightly decreased to 55% in 2021 [16] of total health care expenditures for Cambodian people, which means the patients will need to pay by themselves and the cost is still high. The survey noted that only 20% of the population as a whole might profit from the HEF project [9,13,14]. The other program, the National Social Security Fund (NSSF), was created for the protection and improvement of the well-being of workers in Cambodia [9]. As of 2020, over 1.7 million people were enrolled with the NSSF within over 10 000 businesses that have been registered. Even the HEF and NSSF have been implemented, but most people choose to visit private clinics or hospitals instead of public health care facilities [11–14]. This reflected that the population of Cambodia has become less trusting of public health care due to its limited resources, less treatment capacity, and less effectiveness, both of which still require improvement [1,4].

Devolution of funds to district health societies and decentralisation of planning, implementation, and monitoring can create a sustainable system rooted in provincial and local sociocultural contexts, utilising locally available resources (both human capital and infrastructure). Broad policy frameworks, guidelines, and oversight, however, ought to stay at the federal level. Healthcare decentralisation could increase responsiveness, but it might also make inequities worse. Therefore, the political reforms are crucial for Cambodia’s effective governance; anti-corruption measures and sustained commitment are essential for equitable and successful health system reforms, which are key in preventing gaps in health care [17,18]. The primary health care team’s incentives should be investing in health promotion and prevention, raising health literacy, and cutting costs associated with sickness care. It is recommended that the government promote a comprehensive public health initiative that prioritises health development and inclusive growth as societal goals [15]. Additionally, Cambodia needs to restructure its health care system, giving top priority to the adoption of the ‘Cambodian Public Health Act’ to protect and promote the health of people as a fundamental right to health and health care and the creation of an autonomous ‘Public Health Commission’ to carry out the Act’s mandate. The Public Health Commission should have enough resources (technical staff, equipment, office space, budget, and secretarial support staff) to enable the preparation, funding, implementation, and periodic monitoring of a Public Health Action Plan in collaboration with all relevant sectors to grant everyone the right to a standard and efficient health service to protect people from public health emergencies, improve UHC, and ensure better health and well-being [19]. Innovative in digital health care, including mobile health apps (mHealth), telemedicine, or telehealth, have the potential to enhance Cambodia’s health care system by improving access, reducing costs, and alleviating the burden on health care providers, particularly in rural areas where medical services are limited [20,21]. With high mobile phone penetration, digital health technology can offer remote consultations, support preventive care, and streamline patient management [21]. Effective digital health integration will need Cambodia to make investments in digital health infrastructure, pilot digital health technology initiatives in underprivileged areas, include digital health care in universal coverage plans, and train medical personnel for digital literacy [20,21]. The availability and effectiveness of services might be greatly increased nationwide by prioritising digital health care as part of upcoming health care reforms.

In conclusion, health promotion, disease prevention, and providing health care in a balanced manner are Cambodia’s main challenges in achieving UHC, which will require public policy reforms, strategies, and initiatives across the relevant stakeholders. It is imperative that a multi-sectoral approach be developed and put into action in order to accomplish Sustainable Development Goal Three. The establishment of a Public Health Commission, strengthening the health care worker capacity, and digital health will greatly aid in the coordination of various programs to achieve this improvement in health care service quality and accessibility. This will benefit not only the Ministry of Health but also numerous other relevant ministries and related stakeholders. Furthermore, establishing a responsive governance framework as previously mentioned and allocating 7–10% of gross domestic product to public health [22] are necessary to achieve UHC by 2035.
